# Effect of ethanol extract from *Lactobacillus plantarum* TWK10-fermented soymilk on wound healing in streptozotocin-induced diabetic rat

**DOI:** 10.1186/s13568-019-0886-2

**Published:** 2019-10-11

**Authors:** Yu-Chun Chuang, Meng-Chun Cheng, Chia-Chia Lee, Tai-Ying Chiou, Tsung-Yu Tsai

**Affiliations:** 1 Department of Food Science, Fu Jen Catholic University, No. 510, Zhongzheng Rd., Xinzhuang Dist., New Taipei City, 24205 Taiwan; 2 College of Human Ecology, Fu Jen Catholic University, New Taipei City, Taiwan; 3 Ph. D. Program in Nutrition and Food Sciences, Fu Jen Catholic University, New Taipei City, Taiwan; 4 Culture Collection & Research Institute, SYNBIO TECH INC., Kaohsiung City, Taiwan; 5 School of Regional Innovation and Social Design Engineering, Kitami Institute of Technology, Koen-cho, Kitami, Hokkaido 090-8507 Japan

**Keywords:** Ethanol extract, Wound closure, Diabetes mellitus, *Lactobacillus plantarum* TWK10-fermented soymilk, Skin wounds

## Abstract

Wound healing is a highly dynamic phenomenon comprising numerous coordinated steps including homeostasis/coagulation, inflammation, migration, proliferation, and remodeling. Diabetes mellitus (DM) is a multisystem chronic epidemic that prolongs inflammation in wounds and is associated with impaired healing. This study aimed to investigate the effect of an ethanol extract from *Lactobacillus plantarum* TWK10 (TWK10)-fermented soymilk on wound healing. The anti-inflammatory effects of the ethanol extract of TWK10-fermented soymilk on lipopolysaccharide-stimulated RAW264.7 macrophage cells were examined. The ethanol extract of TWK10-fermented soymilk (100 µg/mL) significantly decreased nitric oxide production from 11.34 ± 0.74 μM to 8.24 ± 2.02 µM (*p* < 0.05) and enhanced proliferation in Detroit 551 cells cultured in high-glucose medium; the cell number peaked at 128.44 ± 7.67% (compared to the untreated control) at 600 µg/mL. An ethanol extract of TWK10-fermented soymilk + vaseline-treated rat model of streptozotocin-induced diabetic wounds was generated herein, and the following groups were formed herein: normal control (NC), blank control (BC), low dose group (LD, 0.24 mg/wound), intermediate dose (MD, 0.48 mg/wound), and high dose (HD, 2.40 mg/wound). On day 14 after wound infliction, the wound area in the LD, MD, and HD groups was significantly decreased to 10.2, 8.4, and 8.5% respectively (*p* < 0.05). Moreover, in the LD, MD, and, HD groups, tumor necrosis factor-α, interleukin 6, and matrix metalloproteinase-9 were downregulated in the wounded skin. These results show that the topical application of the ethanol extract of TWK10-fermented soymilk is beneficial for enhancing wound healing and for the closure of diabetic wounds.

## Introduction

Wound healing is a complex phenomenon involving the repair of damaged skin and other tissues after injury. It involves four complex phases: coagulation, inflammation, proliferation, and remodeling. For efficient wound healing, all four phases and their biophysiological functions must progress sequentially at a specific time with optimal intensity (Lodhi and Singhai 2013). The inflammatory phase plays a key role in wound healing, wherein various growth factors and cytokines that attract macrophages gather at the wound for defense and secrete inflammatory cytokines, which recruit fibroblasts, vascular endothelial cells, epidermal cells, and macrophages to the wound. However, inflammation cannot be decelerated, since it is not conducive to cell migration for wound closure. In the proliferative phase, the wound-surrounding tissue and growth factors and cytokines secreted by macrophages attract fibroblasts to migrate to the injured tissue and begin to proliferate. Thereafter, collagen and glycosides are secreted to constitute an extracellular matrix to accelerate wound healing. Newly generated microvessels form temporary granulation tissue (Clark 1985).

Diabetes is one of the most common chronic metabolic disorders, complications of which are the leading cause of mortality. Oxidative stress can be increased in hyperglycemia, leading to abnormal fibroblast metabolism, thereby reducing cell migration and proliferation and being detrimental to wound healing and inflammation (Shaw et al. 2010). However, excessive oxidative stress in diabetic wounds results in cellular damage and decrease cell differentiation and migration. If keratinocytes and fibroblasts do not migrate successfully to the wound, the healing time and the risk of infection, and consequently limb amputation, are increased (Saltiel and Kahn 2001). A high glucose concentration provides a rich source of nutrients for bacteria growth, thus increasing the risk of traumatic wound infection in patients with diabetes mellitus (American Diabetes 1999).

Soybean is a nutritious food item consumed worldwide, with many traditional phytonutrients and several bioactive phytochemicals including flavonoids, which have various potential health benefits, such as immunomodulatory properties. Soybean can be processed into soymilk, which is an excellent source of nutrients among humans. Soymilk has a nutritional value comparable to that of milk and can be fermented by lactic acid bacteria (Gehrke and Weiser 1947; Patel et al. 1980). Renda et al. reported that isoflavones, genistein and daidzein may potentially promote would healing (Renda et al. 2013). Among isoflavones, genistein, activates macrophages via mitogen-activated protein kinase (MAPK), thus reducing wound pro-inflammatory cytokines (Santos et al. 2013). Moreover, we previously reported that soymilk fermented with *Lactobacillus plantarum* TWK10 significantly increases the levels of daidzein and genistein in ethanol extracts (Cheng et al. 2013). Furthermore, the antioxidant activity of the ethanol extract is greater than that of the water extract (Liu et al. 2016). Mice administered genistein displayed more rapid wound closure probably through a reduction in oxidative stress and modulation of proinflammatory cytokine activity during wound healing (Park et al. 2011).

This study aimed to investigate the effects of the ethanol extract of *Lactobacillus plantarum* TWK10 (TWK10)-fermented soymilk on wound healing. We used lipopolysaccharide (LPS)-induced inflammatory RAW264.7 macrophages and Detroit 551 cells cultured in high-glucose medium and a rat model of streptozotocin-induced diabetic wounds were treated with an ethanol extract of TWK10-fermented soymilk.

## Materials and methods

### Preparation of fermented soymilk and its ethanol extract

The bacterial strain *Lactobacillus plantarum* TWK10 (TWK10) was isolated from Taiwanese fermented cabbage and inoculated at 1% (v/v) for 24–48 h at 37 °C in Lactobacilli deMan, Rogosa and Sharpe (MRS) broth. It has been deposited in depository, Bioresource Collection and Research Center, Food Industry Research and Development Institute (HsinChu, Taiwan), and given accession number of BCRC 910734. Soymilk was prepared as described previously (Cheng et al. 2013). Soybeans were soaked in water for 8 h at 25 °C and the swollen soybeans were homogenized with water. The resulting slurry was filtered through a sieve and heated at 90 °C for 1 h in a water bath to obtain soymilk. Each 100-g fraction of soymilk contains 6.1 g of protein, 2.8 g fat, 0.4 g saturated fat, 2.1 g carbohydrate, and 6 mg sodium. All experimental steps were carried out in a laminar air flow, and sterilized soymilk was inoculated 1% TWK10 bacteria and incubated at 37 °C for 48 h. Thereafter, fermented soymilk was freeze-dried. The ethanol extract of TWK10-fermented soymilk was prepared with 40 g dried soymilk powder and 170 mL ethanol at 37 °C in a water bath for 30 min, and then placed in a 37 °C shaker incubator for 30 min at 150 rpm. After centrifugation at 12,000×*g* for 30 min at 4 °C, the supernatant was concentrated under reduced pressure and lyophilized to obtain the fermented soymilk ethanol extract.

### NO production by RAW264.7 cells and proliferation in Detroit 551 cells by upon treatment with the ethanol extract of TWK10-fermented soymilk

Murine macrophage cell line (RAW264.7) BCRC 60001 and human skin fibroblasts (Detroit 551) BCRC 60118 were obtained from the Bioresource Collection and Research Centre, Food Industry Research and Development Institute (Hsinchu, Taiwan). RAW264.7 mice macrophages (1 × 10^4^ cells/well) were inoculated in 96-well plates and cultured at 37 °C for 24 h. The supernatant was discarded and samples were co-incubated with LPS for 24 h. Hundred microliters of the supernatant was treated with 100 μL of Griess reagent at 25 °C for 5 min. The absorbance was determined at 550 nm with NaNO_2_ used as the standard, and plotted in a regression curve to determine the levels of NO released by RAW264.7 cells. In addition, Detroit 551 human skin fibroblasts (1 × 10^3^ cells/well) were inoculated in 96-well plates and cultured at 37 °C for 24 h. After discarding the supernatant, media with different glucose content and different sample concentrations were co-incubated for 24 h. On discarding the supernatant again, 200 μL of MTT reagent was added and the plates were incubated at 37 °C for 1 h; thereafter, the supernatant was discarded and 200 μL DMSO was added to dissolve the purple crystals. The absorbance was then determined at 550 nm, using an ELISA reader.

### Wound healing in rats upon treatment with the ethanol extract of TWK10-fermented soymilk

Thirty-six Wistar rats (9-week-old, 300 g, BioLASCO Taiwan Co., Ltd., Taipei, Taiwan) were housed at the Fu Jen Laboratory Animal Center (Taipei, Taiwan) at 21 ± 2 °C and 55 ± 10% relative humidity with a 12:12-h light/dark cycle. Rats were fasted for more than 12 h before induction, followed by intraperitoneal injection of STZ (65 mg/kg body weight, citric acid buffer pH 4.5). The rats were divided into 6 groups (n = 6 rats each) (Table 1). All the rats were subjected to induced diabetes and wounds were inflicted, except for the normal control (NC) group, wherein only the wound was induced. The blank control (BC) group was considered the diabetes control group and the wound without treatment. The positive control (PC) group was treated with Suile^®^ (bismuth subgallate 4.5%, borneol 0.7%, and petrolatum 94.8%) as the standard. The test groups had 3 doses: a low dose (LD, 0.24 mg/wound), intermediate dose (MD, 0.48 mg/wound), and high dose (HD, 2.40 mg/wound). Genistein was used as an indicator (15.9 μg/cm^2^) in the MD group in accordance with our previous studies (unpublished). Fasting blood glucose was measured in blood sampled from the tail vein. When the fasting blood glucose of the rats exceeded 200 mg/dL, the rats were considered to have diabetes and were ready for wound infliction. The rats were anesthetized with isoflurane, and 75% alcohol was used to disinfect the dorsal surface of the rats and surgical instruments, and fur was shaved off. A drilling round knife was used to inflict six wounds on dorsal surface, each with a 0.8-cm diameter and the depth of the lipid layer (Hozzein et al. 2015). After the wounds were inflicted and wrapped, they were treated with the ethanol extract of TWK10-fermented soymilk + vaseline through topical application at dosages of 0.24, 0.48, and 2.40 mg/wound daily. The wound area was then measured on days 1, 4, 7, 10, and 14 of treatment. The percent (%) wound was calculated using the following formula: wounded area (%) = detected wound area/Initial wound area × 100%. The animal experimental protocol was reviewed and approved by the Institutional Animal Care and Use Committee of the Fu Jen Catholic University (IACUC Approved No: A10506).

**Table 1:** Experimental animals and grouping

Groups	Dose of sample (mg/wound)	Wound treatment
NC	–	Untreatment
BC	–	Untreatment
PC	–	Treatment with Suile^®^
LD	0.24	Treatment with low dose of ethanol extract from TWK10-fermented soy milk
MD	0.48	Treatment with middle dose of ethanol extract from TWK10-fermented soy milk
HD	2.40	Treatment with high dose of ethanol extract from TWK10-fermented soy milk

NC: normal control; BC: blank control; PC: wounded STZ-induced diabetic rats treatment with Suile^®^; LD: wounded STZ-induced diabetic rats treatment with low dose ethanol extract from TWK10-fermented soymilk. MD: wounded STZ-induced diabetic rats treatment with middle dose ethanol extract from TWK10-fermented soymilk. HD: wounded STZ-induced diabetic rats treatment with high dose ethanol extract from TWK10-fermented soymilk. STZ: streptozotocin

### Inflammatory cytokine and protein expression in the wounded skin

All animals were fasted for 16 h before they were euthanized via carbon dioxide inhalation (day 4 and day 14). Blood was sampled, and serum and plasma samples were obtained, as previously reported (Liu et al. 2016). Skin samples were harvested on day 4 and day 14 after wounding and divided into two parts: one formalin-fixed paraffin-embedded part subsequently subjected to hematoxylin and eosin staining (Wang et al. 2017) and the other part was homogenized thrice with a Nonidet P-40/SDS lysis buffer (200 μL, 1% Nonidet P-40, 0.01% SDS, 0.1 M Trizma^®^ hydrochloride at pH 7.2, 100 mM phenylmethanesulfonyl fluoride, and 1 mg/mL aprotinin), using a FastPrep^®^ System (MP Biomedicals, Santa Ana, CA, USA), with overnight aging. The lysate was centrifuged at 12,000×*g* for 30 min at 4 °C, and the supernatant was harvested. Thereafter, total protein lysate (40 μg) was resolved via SDS-polyacrylamide gel electrophoresis (PAGE; 7.5% resolving gel) and subsequently electro-transferred onto a polyvinylidene difluoride membrane. Thereafter, the membranes were blocked with Tris-buffered saline containing 2% non-fat milk powder and were incubated overnight in blocking solution containing rat anti- tumor necrosis factor-α (TNF-α) antibody (1:2500), rat anti- interleukin-6 (IL-6) antibody (1:2500), rat anti-matrix metalloproteinase 9 (MMP-9) antibody (1:1000), and mouse anti-β-actin (1:5000). The blots were then incubated with blocking buffer containing the corresponding horseradish peroxidase (HRP)-conjugated secondary antibodies for 1 h at 25 °C. Protein bands were visualized using an enhanced chemiluminescence kit (Amersham Pharmacia Biotech, Arlington Heights, IL, USA) and a UVP image analysis system (UVP, Upland, CA, USA). The analysis was performed using the Gel-Pro Analyzer 4 (Media Cybernetics, Inc., Rockville, MD, USA).

### Statistical analysis

Data are represented by mean ± standard deviation values and analyzed using the Statistical Package for the Social Sciences software (SPSS for Windows, version 10.0.7C, SPSS Inc., Chicago, IL, USA). Differences in the effect of treatment were analyzed using one-way ANOVA followed by Duncan’s multiple range test. Each experiment was performed in triplicate, and the significance level was set at *p* < 0.05.

## Results

### Effect of the ethanol extract of TWK10-fermented soymilk on NO production in RAW264.7 cells and cell proliferation in Detroit 551 cells

**Fig. 1:**
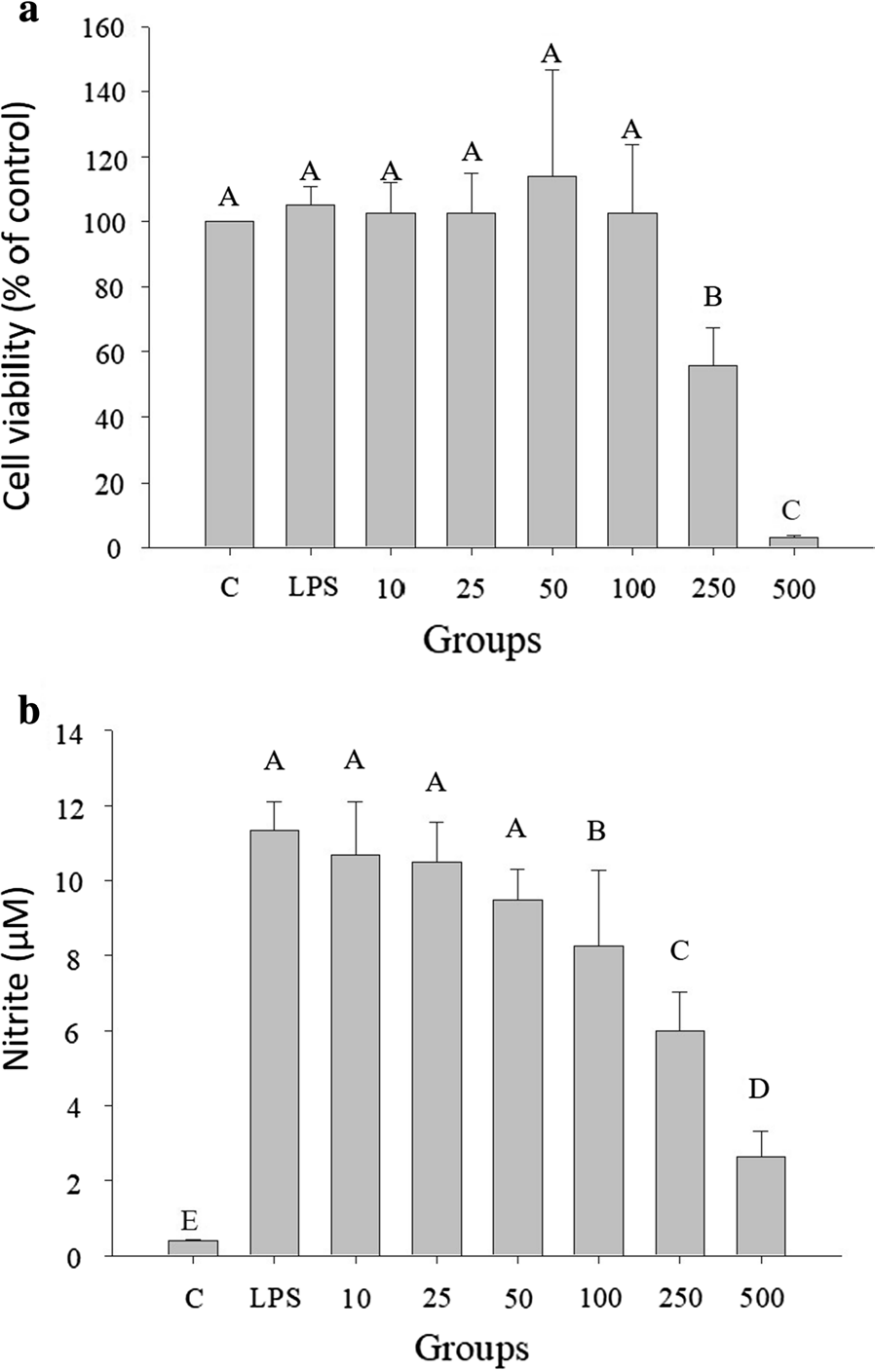
Effects of TWK10-fermented soymilk ethanol extract on **a** cell viability and **b** NO production of lipopolysaccharide-induced inflammation RAW264.7 macrophages. Cells were treated with various concentrations of TWK 10-fermented soy milk ethanol extract (10–500 µg/mL) for 24 h. Cell viability was measured by MTT assay and were represented as % of control cell viability. The data are presented as mean ± SD (n = 3). Values with different uppercase letters were significantly by Duncan’s multiple range tests (*p* < 0.05)

Tolerant concentrations of RAW264.7 cells induced with LPS upon treatment with ethanol extracts of TWK10-fermented soymilk are shown in Fig. 1. The viability of RAW264.7 cells induced with LPS was 94.29 ± 8.24–109.75 ± 30.89% at 10–100 μg/mL. However, cell viability decreased to 50.43 ± 12.63% and 3.53 ± 1.04% at 250 and 500 µg/mL, respectively. The ethanol extracts of TWK10-fermented soymilk at 100 μg/mL were used as the maximum concentration to treat RAW264.7 cells. NO production in RAW264.7 cells was significantly decreased from 11.34 ± 0.74 µM to 8.24 ± 2.02 µM at 100 μg/mL of ethanol extracts of TWK10 fermented soymilk in comparison with the LPS-induced group.

**Fig. 2:**
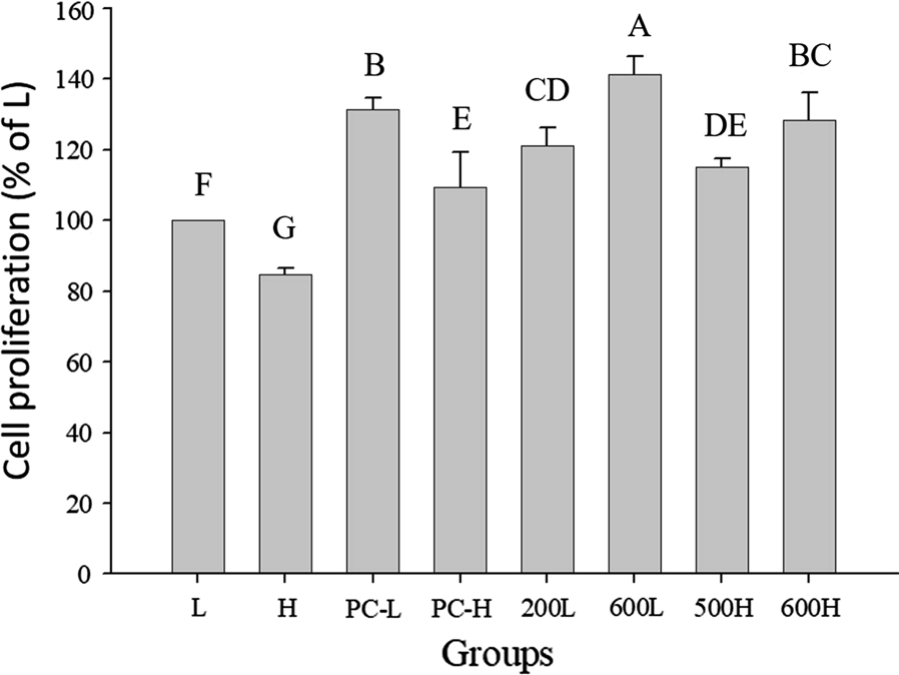
Effect of the ethanol extract of TWK10-fermented soy milk on cell proliferation of Detroit 551 cells culture in the high and low glucose medium. Cell proliferation was measured by MTT assay and were represented as % of cells culture in the low glucose medium. The data are presented as mean ± SD (n = 3). Values with different uppercase letters were significantly by Duncan’s multiple range tests (*p* <  0.05). L: cells culture in the low glucose medium; H: cells culture in the high glucose medium. PC-L and PC-H: cells culture with all-trans retinoic acid in the low and high glucose medium. 200L and 600L: cells culture with TWK10-fermented soy milk ethanol extract in the low glucose medium. 500H and 600H: cells culture with TWK10-fermented soy milk ethanol extract in the high glucose medium

Human skin fibroblasts, Detroit 551 cells, were co-cultured with the sample in low-glucose (5 mM) medium (Fig. 2). The cell proliferation rate in low-glucose medium (5 mM) was significantly increased to 121.11 ± 5.21 and 141.32 ± 5.11% at 200 and 600 μg/mL, respectively, in comparison with the control group (*p* < 0.05). Furthermore, cell proliferation in Detroit 551 cells cultured in high-glucose medium (25 mM) and treated with ethanol extracts of TWK10 fermented soymilk was significantly increased to 115.18 ± 2.23% and 128.44 ± 7.67% at 500 and 600 μg/mL, respectively, in comparison with the control group (*p* < 0.05). In summary, ethanol extracts of TWK10-fermented soymilk increased the proliferation in Detroit 551 cells cultured in both low- and high-glucose media.

### Fasting serum glucose

Before STZ-induced diabetes, no significant difference was observed in fasting glucose levels in all groups (Table 2). After STZ administration, rats with diabetes presented a 3- to 4-fold increase in fasting glucose levels, which were significantly higher than those of the control group (*p* < 0.05). During the experimental period, fasting glucose levels remained steady in rats with diabetes. Therefore, topical treatment of rat wounds did not elicit any effect on the fasting glucose level.

**Table 2:** The fasting serum glucose of wounded STZ-induced diabetic rats

Groups	Fasting serum glucose (mg/dL)						
	Before induced diabetes	Before wounded	Day 1 post-wounded	Day 4 post-wounded	Day 7 post-wounded	Day 10 post-wounded	Day 14 post-wounded
NC	110.9 ± 11.7^Ab^	118.1 ± 10.3^Bb^	124.8 ± 16.2^Bb^	116.1 ± 9.8^Cb^	115.5 ± 7.6^Bb^	120.3 ± 13.2^Bb^	167.1 ± 18.0^Ba^
BC	112.2 ± 10.2^Ac^	360.5 ± 80.0^Ab^	398.4 ± 31.6^Aab^	394.0 ± 60.0^ABab^	424.5 ± 16.0^Aa^	449.2 ± 13.1^Aa^	399.6 ± 43.2^Aab^
PC	105.0 ± 9.0^Ac^	395.3 ± 36.7^Aab^	367.6 ± 58.2^Ab^	446.8 ± 71.6^Aa^	381.0 ± 72.8^Aab^	416.7 ± 50.3^Aab^	408.7 ± 59.0^Aab^
LD	115.6 ± 8.3^Ab^	429.2 ± 60.8^Aa^	389.5 ± 28.1^Aa^	371.7 ± 56.7^Ba^	412.1 ± 40.0^Aa^	429.4 ± 16.8^Aa^	411.4 ± 55.5^Aa^
MD	113.6 ± 9.2^Ab^	372.0 ± 63.9^Aa^	377.8 ± 30.5^Aa^	389.2 ± 33.7^ABa^	415.8 ± 47.4^Aa^	417.6 ± 55.1^Aa^	432.7 ± 70.7^Aa^
HD	107.1 ± 15.6^Ad^	367.9 ± 40.1^Ac^	369.7 ± 21.7^Ac^	401.7 ± 25.4^ABbc^	422.6 ± 58.8^Aab^	450.8 ± 41.1^Aa^	402.0 ± 39.8^Abc^

The data are presented as mean ± SD (n = 6). Values with different uppercase letters were significantly different in the same column and values with different lowercase letters were significantly different in the same row by Duncan’s multiple range tests (*p* < 0.05). NC: normal control; BC: blank control; PC: wounded STZ-induced diabetic rats treatment with Suile^®^; LD: wounded STZ-induced diabetic rats treatment with low dose ethanol extract from TWK10-fermented soymilk. MD: wounded STZ-induced diabetic rats treatment with middle dose ethanol extract from TWK10-fermented soymilk. HD: wounded STZ-induced diabetic rats treatment with high dose ethanol extract from TWK10-fermented soymilk. STZ: streptozotocin

### In vivo wound healing experiments

**Fig. 3:**
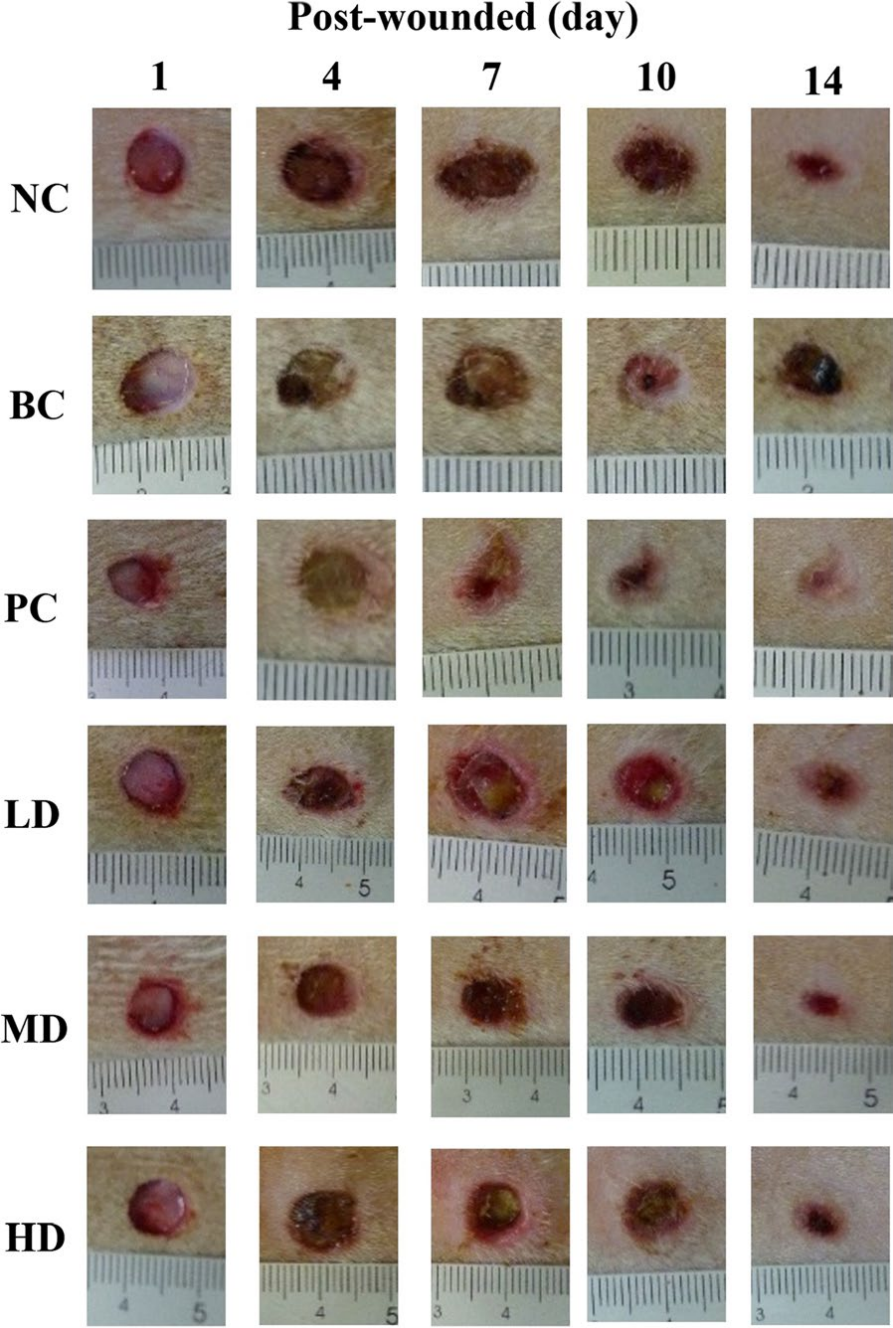
Skin excisional wound area of wounded STZ-induced diabetic rats. NC: normal control; BC: blank control; PC: wounded STZ-induced diabetic rats treatment with Suile^®^; LD: wounded STZ-induced diabetic rats treatment with ethanol extract from TWK10-fermented soy milk at the dose of 0.24 mg/wound. MD: wounded STZ-induced diabetic rats treatment with ethanol extract from TWK10-fermented soy milk at the dose of 0.48 mg/wound. HD: wounded STZ-induced diabetic rats treatment with ethanol extract from TWK10-fermented soy milk at the dose of 2.40 mg/wound

Figure 3 shows the comparison in the wound healing process on different days in the experimental groups. In rats treated with the ethanol extract of TWK10-fermented soymilk + vaseline, healing was faster than that in the control group, thus showing remarkable differences in the wound area and morphology during wound healing (day 10 and day 14). The wound area was quantitated as a percentage relative to the wound area on day 1 of wound infliction (Table 3). The wound area of normal control (NC) and blank control (BC) groups differed significantly during wound healing (*p* < 0.05). Rats with diabetes presented slower wound healing than the NC group. Moreover, in LD, MD, and HD groups, at the early stage (day 4), distinct differences were observed in the wound area. At the intermediate stage, at approximately day 7 to day 10, the wound area decreased significantly from 55 to 22% and remained stable at approximately 8–10% on day 14 (*p* < 0.05). These results show that treatment with the ethanol extract of TWK10-fermented soymilk can enhance wound healing in rats with STZ-induced diabetic trauma.

**Table 3:** Effect of treatment with ethanol extract from TWK10-fermented soymilk on macroscopic changes at skin excisional wound sites of wounded STZ-induced diabetic rats

Groups	Wounded area (%)				
	Day 1 post-wounded	Day 4 post-wounded	Day 7 post-wounded	Day 10 post-wounded	Day 14 post-wounded
NC	100.0 ± 17.2^Aa^	78.0 ± 22.2^Cb^	40.7 ± 16.6^Cc^	19.8 ± 8.1^Cd^	4.1 ± 5.3^Be^
BC	100.0 ± 23.2^Ab^	112.2 ± 38.4^Aa^	84.6 ± 33.3^Ab^	50.9 ± 26.8^Ac^	26.1 ± 24.5^Ad^
PC	100.0 ± 23.0^Aa^	89.7 ± 9.3^BCb^	56.1 ± 15.4^Bc^	28.2 ± 17.2^BCd^	10.0 ± 5.9^Be^
LD	100.0 ± 23.5^Aa^	92.6 ± 23.7^Bb^	55.6 ± 19.3^Bc^	29.5 ± 16.0^Bd^	10.2 ± 8.0^Be^
MD	100.0 ± 21.5^Aa^	83.8 ± 30.8^BCb^	47.1 ± 16.8^BCc^	23.4 ± 9.9^BCd^	8.4 ± 7.4^Be^
HD	100.0 ± 19.9^Aa^	90.5 ± 26.1^BCb^	50.7 ± 16.2^BCc^	22.1 ± 11.4^BCd^	8.5 ± 4.5^Be^

The data are presented as mean ± SD (n = 6). Values with different uppercase letters were significantly different in the same column and values with different lowercase letters were significantly different in the same row by Duncan’s multiple range tests (*p* < 0.05). NC: normal control; BC: blank control; PC: wounded STZ-induced diabetic rats treatment with Suile^®^; LD: wounded STZ-induced diabetic rats treatment with low dose ethanol extract from TWK10-fermented soymilk. MD: wounded STZ-induced diabetic rats treatment with middle dose ethanol extract from TWK10-fermented soymilk. HD: wounded STZ-induced diabetic rats treatment with high dose ethanol extract from TWK10-fermented soymilk. STZ: streptozotocin

### Protein expression in wounded tissue

**Fig. 4:**
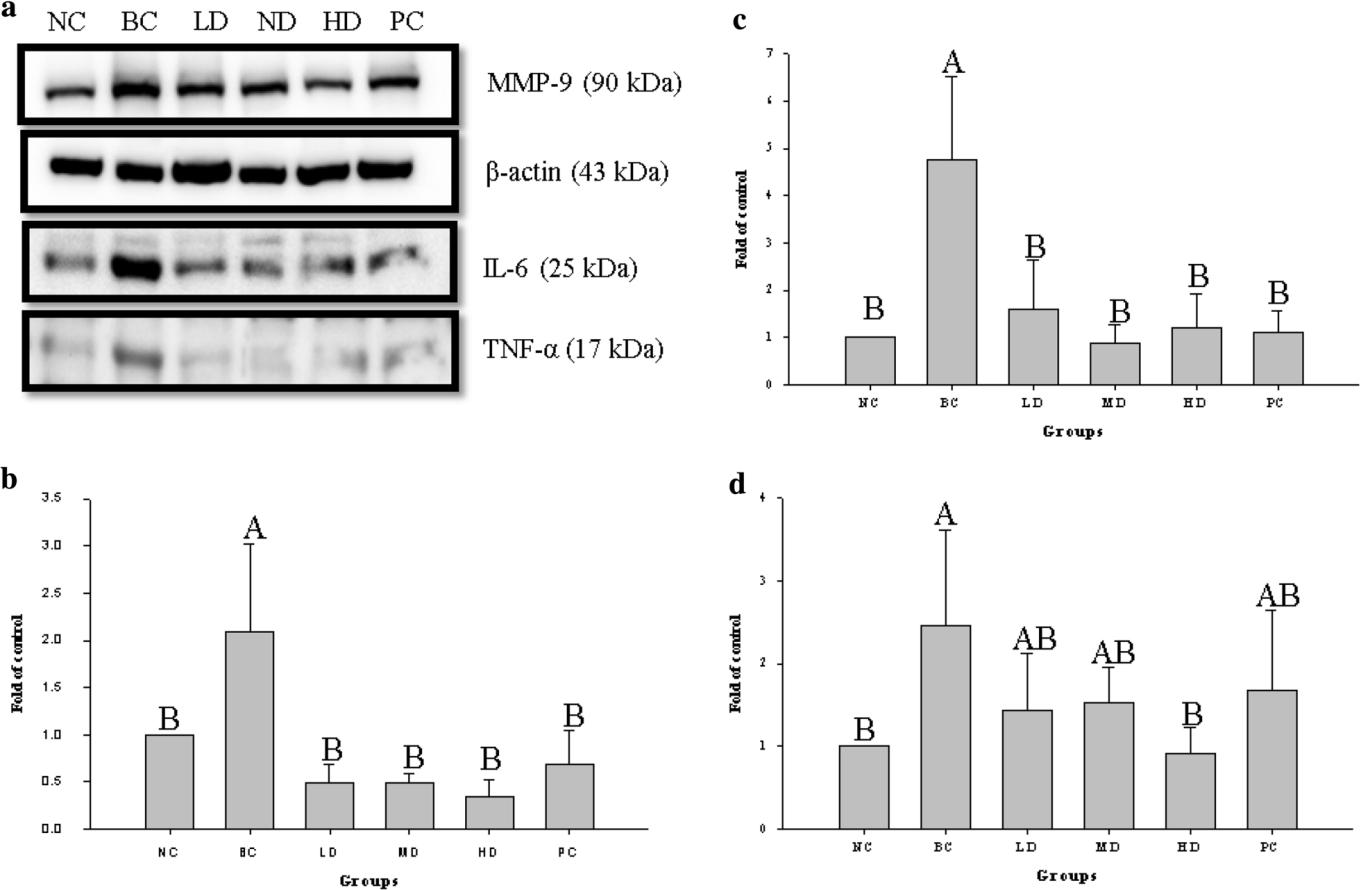
Effect of treatment with ethanol extract from TWK10-fermented soy milk on the **a** expression of protein in skin of wounded STZ-induced diabetic rats 4 day post-wounded; **b** TNF-α; **c** IL-6; **d** MMP-9. The data are presented as mean ± SD (n = 3). Values with different uppercase letters were significantly different by Duncan’s multiple range tests (*p* < 0.05). NC: normal control; BC: blank control; PC: wounded STZ-induced diabetic rats treatment with Suile^®^; LD: wounded STZ-induced diabetic rats treatment with low dose ethanol extract from TWK10-fermented soy milk. MD: wounded STZ-induced diabetic rats treatment with middle dose ethanol extract from TWK10-fermented soy milk. HD: wounded STZ-induced diabetic rats treatment with hight dose ethanol extract from TWK10-fermented soy milk. STZ: streptozotocin

**Fig. 5:**
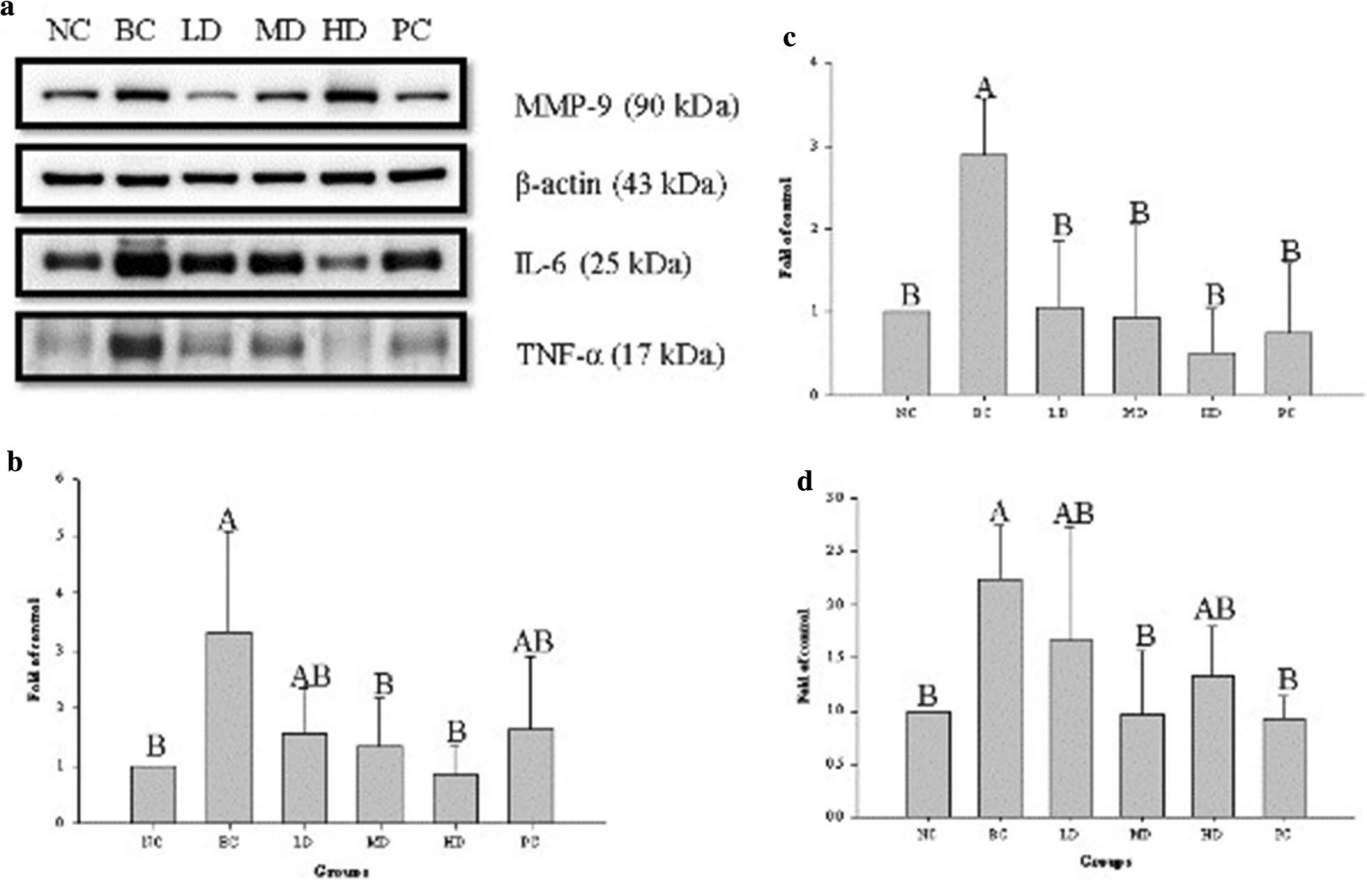
Effect of treatment with ethanol extract from TWK10-fermented soy milk on the **a** expression of protein in skin of wounded STZ-induced diabetic rats 14 day post-wounded; **b** TNF-α; **c** IL-6; **d** MMP-9. The data are presented as mean ± SD (n = 3). Values with different uppercase letters were significantly different by Duncan’s multiple range tests (*p * < 0.05). NC: normal control; BC: blank control; PC: wounded STZ-induced diabetic rats treatment with Suile^®^; LD: wounded STZ-induced diabetic rats treatment with low dose ethanol extract from TWK10-fermented soy milk. MD: wounded STZ-induced diabetic rats treatment with middle dose ethanol extract from TWK10-fermented soy milk. HD: wounded STZ-induced diabetic rats treatment with hight dose ethanol extract from TWK10-fermented soy milk. STZ: streptozotocin

To analyze TNF-α, IL-6, and MMP-9 expression during wound healing, we analyzed traumatized skin on day 4 and day 14 after wounding. As shown in Figs. 4 and 5, TNF-α, IL-6, and MMP-9 were significantly upregulated in traumatized skin of rats with STZ-diabetes than in normal trauma rats (NC group) (*p* < 0.05). On day 4 and day 14 after wounding, TNF-α and IL-6 were significantly downregulated in MD and HD groups compared with the BC group (*p* < 0.05). Moreover, MMP-9 was downregulated in the in LD, MD, and HD groups than in the BC group.

### Collagen deposition

**Fig. 6:**
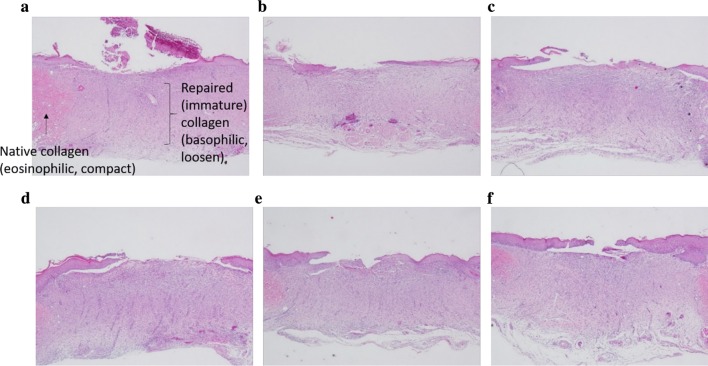
Treatment with ethanol extract from TWK10-fermented soy milk on histopathological findings of skin wound on day 10 in rats. Skin wound showed minimal to moderate inflammation, minimal to slight angiogenesis, moderate to very thick granulation layer, and re-epithelialization was graded as little in NC (**a**), moderate in BC (**b**), little in PC (**c**), moderate in LD (**d**), MD (**e**), and HD (**f**). H&E stain. ×40

**Fig. 7:**
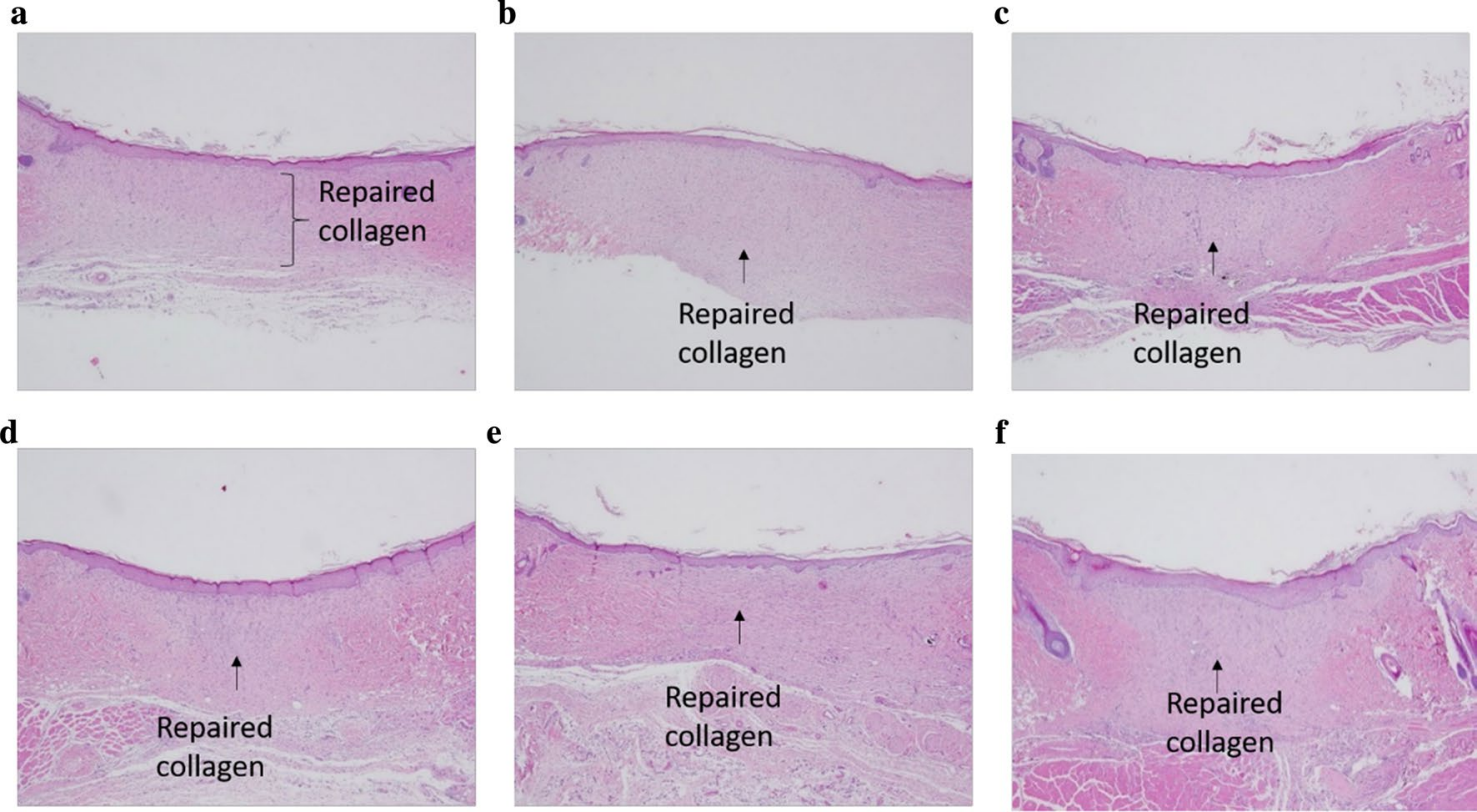
Treatment with ethanol extract from TWK10-fermented soy milk on histopathological findings of skin wound on day 14 in rats. Skin wound showed absent inflammation and angiogenesis, very thick granulation layer, and re-epithelialization was graded as complete in NC (**a**), BC (**b**), PC (**c**), LD (**d**), MD (**e**), and HD (**f**). H&E stain. ×40

The results of histological analysis of traumatized tissue, via H&E staining, are shown in Figs. 6 and 7. Some fibroblasts and inflammatory cell infiltration into the upper layers of dermis were observed at the wound 10 d after surgery (Fig. 6). Furthermore, collagen fibers were formed at this point in the NC and HD groups. In addition, deposition of collagen fibers was clearly observed on day 14 after wounding in the NC, PC, LD, MD, and HD groups, other than the BC group (Fig. 7). Overall, the ethanol extract of TWK10-fermented soymilk + vaseline-treated groups displayed slightly but not significantly higher efficacy of collagen fiber formation in comparison with the BC group.

## Discussion

MAPK plays a major role in cell proliferation, differentiation, inflammatory, and apoptosis (Zhang and Liu 2002). Treatment with MAPK inhibitors (SCIO-469, PD-98059, and PD98059) accelerated wound healing (Draper et al. 2003; Lima et al. 2012; Medicherla et al. 2009), suggesting that the MAPK pathway markedly promotes wound closure. The ethanol extracts of TWK10-fermented soymilk displayed anti-inflammatory effects and enhanced the proliferation of human skin fibroblasts in vitro. UVB irradiation stimulated the cell mixtures (keratinocytes and fibroblasts) to secrete pro-inflammatory cytokines via mitogen-activated protein kinase (MAPK) signaling pathways. Soybean isoflavonoids (daidzin, daidzein, genistin, and genistein) regulated the MAPK signaling cascade. In particular, genistein treatment potently inhibited IL-6 production and MAPK signaling than other isoflavonoids (Lee et al. 2014). Kang et al. (2007) reported that equol potentially suppresses LPS-induced NO production and iNOS expression by intercepting Akt activation and subsequently downregulating NF-κB (Kang et al. 2007). The β-glucosidase enzyme from lactic acid bacteria in soy milk hydrolyzes isoflavone glucosides (daidzin and genistin) to aglycones, daidzein and genistein (Rekha and Vijayalakshmi 2011). While daidzein s converted to equol via the formation of the intermediate dihydrodaidzein (Rafii 2015), during wound repair, blood reperfusion and angiogenesis contribute to tissue hypoxia during inflammation. The inflamed tissue commonly displays hypoxia and concomitantly increases the production of free radicals and reactive oxygen species at the wound, thus causing improper or prolonged wound healing (Woo et al. 2007). Hyperglycemia causes oxidative stress, which causes the antioxidant capacity to be exceeded and promotes inflammation (Vincent et al. 2004). Therefore, the inflammatory phase is a critical stage during wound healing. We previously reported that daidzein and genistein levels were greater in soymilk fermented with TWK10 than in *Streptococcus thermophilus* BCRC14085 and *Pediococcus pentosaceus* MK6 after 48 h incubation (Chen et al. 2013; Cheng et al. 2013). Otieno et al. (2006) reported that the bioconversion rate of aglycone isoflavones in fermented soymilk is less than 50% after 6 h. We previously reported that in approximately 93.87% of cases, daidzein and genistein levels were 143.51–144.34 and 75.71–113.08 μg/mL, respectively (Chen et al. 2013; Cheng et al. 2013). Equol was observed upon treatment with the ethanol extract of TWK10-fermented soymilk, being approximately 1.48- and 0.54-fold those of daidzein and genistein, respectively in our previous analysis. Together, we considered that the isoflavonoids (daidzein, genistein, and equol) are the functional components of TWK10-fermented soymilk.

Hyperglycemia is associated with abnormal fibroblast function (American Diabetes 1999). High glucose levels inhibit cell proliferation and migration and angiogenesis, and enhanced cell apoptosis results in prolonged wound healing (Lorenzi et al. 1987). Following injury, fibroblasts in the surrounding tissue are stimulated to proliferate. On inflicting the wound, fibroblasts proliferate profusely with increased production of collagen, hydroxyproline, hexose, and hyaluronic acid. Moreover, consistent with angiogenesis, granulation tissue formation and remodeling occur. The release of cytokines and chemokines accelerate epidermal cell migration and proliferation in wounds (Barry 2000; Haas and Grekin 1995; Ponrasu and Suguna 2014; Wagner and Wehrmann 2007). Therefore, fibroblasts play a vital role in cutaneous wound healing.

Methylglyoxal (MGO) is considered a major precursors of advanced glycation end products (AGEs), which eventually delay epithelial wound healing in diabetic skin (Ge et al. 2013), indicating that food items rich in flavonoids can prevent various diabetic complications (Shao et al. 2014). Genistein significantly reduced MGO and AGE levels in vivo via multiple pathways, e.g., by trapping AGEs (Rahman Mazumder and Hongsprabhas 2016; Zhao et al. 2019). Excess formation of AGEs is considered the prominent pathomechanism in diabetes. AGEs are produced alter the formation of the new extracellular matrix and cytokines and induce cellular function (American Diabetes 1999). Treatment of human skin organ cultures with soybean extract increases fibroblast proliferation (Varani et al. 2004). Our results show that ethanol extracts of TWK10-fermented soymilk stimulate proliferation in Detroit 551 cells in both low- and high-glucose media (Fig. 2). During wound healing, there numerous inflammatory cytokines contribute to excessive scarring, including hypertrophic scars (HS), a dermal manifestation of fibroproliferative disorders. Excessive collagen deposition is implicated in HS pathogenesis. HS commonly occurs owing to injuries to the deep dermis, including trauma, burn injury, abrasions, and surgery (Thompson et al. 2013). Furthermore, HS can cause significant esthetic and functional abnormalities (Serghiou et al. 2009). Varani et al. (2004) reported that treatment with genistein (0.5 and 1.0 μg/mL) did not influence cell proliferation in organ cultures of human skin (Varani et al. 2004). However, genistein inhibits the proliferation and function of hypertrophic scar fibroblasts and type I and III collagen levels (Cao et al. 2009). High MMP-9 activity may alter collagen synthesis and release cytokines at the site of injury. Therefore, the expression of MMP-9, TNF-α, and other growth factors is a potential therapeutic target (Ayuk et al. 2016). Our study shows that continuous topical application of ethanol extracts of TWK10-fermented soymilk not only downregulates pro-inflammatory cytokines (TNF-α and IL-6) but also regulates MMP-9 protein levels. Although this study shows the promising effects of the ethanol extract of TWK10-fermented soymilk on wound healing in a rat model of streptozotocin-induced diabetes, the underlying mechanism is unclear; hence, future studies are required to examine the mechanism of action of the ethanol extract of TWK10-fermented soymilk in detail, along with the bioactive compounds in the extract.

Together, these results show the wound healing efficacy of ethanol extracts of TWK10-fermented soymilk in diabetes rats through a reduction in the inflammatory response, stimulation of cell proliferation, and formation of the extracellular matrix. In future, The ethanol extract of TWK10-fermented soymilk can be further applied as material for dressing wounds to promote diabetic wound healing.

## Data Availability

Data will be shared whenever it is required.

## Abbreviations

AGEs: advanced glycation end products
DM: diabetes mellitus
HS: hypertrophic scars
IL-6: interleukin 6
LPS: lipopolysaccharide
MAPK: mitogen-activated protein kinase
MMP-9: matrix metalloproteinase-9
NO: nitric oxide
STZ: streptozotocin
TNF-α: tumor necrosis factor-α
TWK10: *Lactobacillus plantarum* TWK10
